# The Virtuous Health Workers

**DOI:** 10.12669/pjms.36.COVID19-S4.2733

**Published:** 2020-05

**Authors:** Shabih H. Zaidi

COVD-19 has brought the world to its knees. People are already comparing it to the Spanish flu of 1918, and some have even gone to mention the Biblical plague of Antonine plague of 165-180AD, when even Galen is said to have fled the scene.^1^

Never mind the developing nations, even the lords and masters of the world like USA, China, EU and U.K. are in a state of dire emergency, lock down and of course rising number of fatalities. Economies have collapsed and no one, not even the sages and savants of financial world can see a light at the end of the tunnel.

A new world order will emerge for those who survive this deluge of Noah. One hopes the exploitation of the poor nations by the rich and powerful nations would have perished with the deadly virus; so that enough resources could be used by the governments in serving mankind not annihilating them. Instead of talking about services within limited resources one may be talking of social justice and provision of equal playing fields to one and all.

While the politicians struggle with major administrative decisions, and the economist worrying about the world beyond the pandemic, we as health care workers are facing the real onslaught of the disease. There is not a single person who may not have either lost a friend or about to lose one to the pandemic while saving others’ lives. 2.56 million are already affected globally as reported by Johns Hopkins^2^ with over 157000 deaths, and many more still feared to die.

British NHS is a typical example which has arguably failed to deliver. 60 health workers have died, and numerous colleagues are currently on ventilators, or downgraded into HDUs, continuing to suffer. Rest of the nation is locked down.

Politician continue to debate that enough equipment’s and supplies are available to frontline health workers. But that is not true. Several of my acquaintances in the NHS have failed to receive or obtain the gowns, the goggles, or the gloves. Some have been donated the lab goggles by schools, and others are using head scarves and other makeshift face masks, which are absolutely unsatisfactory to say the least.

On 5 April, 2020, a major news channel asked the health secretary about the shortage of drugs and medicines based upon a rumour that USA was attempting to takeaway large component of such drugs depriving the U.K. of its share. New York is in a state of free fall. Major hospitals like Mount Sianai and Columbia Presbyterian are facing acute crisis of shortage of beds and staff. Similar situation is seen in Atlanta and California.

The question here is not of the number of victims etc, but a more important ethical question of duty of care. Physicians are sworn to serve the sick, at all times. But does the oath also say that one is obliged to serve the sick even at the cost of ones’ own life? Hippocratic Oath doesn’t. It ends with these words ‘Now if I carry out this oath, and break it not, may I gain for ever reputation among all men for my life and for my art; but if I break it and forswear myself, may the opposite befall me. Translation by W.H.S. Jones’.^3^

History informs us that during the monumental plagues many physicians abandoned the cities and societies to save their own lives. It has not happened so far. Nurses, doctors and paramedics are dying in nearly even country, and are standing fast, to save human lives.

Ethical question needs to be discussed on the basis of established Western theories, though one may not always agree with all or even some of them. But since we practice medicine following the established norms which are. (a) Deontology (b) Utilitarianism and (c) Virtue ethics. So let us evaluate the current situation in the light of these theories.

***Deontology*** is based upon Kantian philosophy of Duty and Reasoning. So, a health worker is duty bound to serve the sick as a call of duty.

Utilitarianism or Consequestionalism is based upon John Stewart Mills’ theory of working for the goodness of larger number of people with a view to maximise the benefits. So one may eliminate a robber or a murderer to save many others in a community.

***Virtue ethics*** was introduced by Plato, and augmented by Aristotle, identifying four cardinal principles m namely courage, compassion, practical wisdom and justice. It went into eclipse for a thousand years or more, and has only recently gained revival through McIntyre, and Pellegrino. Virtue ethics focuses on character building.

***‘Virtue*** Ethics. Virtue Ethics (or Virtue Theory) is an approach to Ethics that emphasises an individual’s character as the key element of ethical thinking, rather than rules about the acts themselves (Deontology) or their consequences (Consequentialism)’.^4^

Now if we examine all these theories of Ethics, in the light of Islamic teaching, we may find that all of them merit certain values, though one may differ in the order of priority is Autonomy, Beneficence, Non maleficence and Justice. Cultural Relativism but not Moral Relativism, is accepted by nearly all. Moral values are often culture and faith dependent. eg: polygamy, shared motherhood, tattooing, transgenderism and so forth accepted by some and condemned by others.

Islam concentrates on character building. The fundamental concept of Virtue ethics is to build a character. So, for instance Deontology will expect that if you see a blind man crossing the road, you would volunteer to help him cross.

Well, what we see is that the moment an emergency was declared, health workers volunteered to serve the sick despite the risks and threat to their personal life. A retired surgeon in the Midlands jumped to share the burden of care as soon as he heard the call, all hands to over to deck. Sadly, he succumbed. A renowned dental surgeon, a senior GP, an internist and several nurses have volunteered and eventually ended up on ventilators or passed away, while saving others.

Similar picture is witnessed in Pakistan, where numerous health professionals have gone out of the way to serve not just in designated hospitals but even to remote mountainous regions like Gilgit and Baltistan, where one brave young colleague Dr. Osama, succumbed. Medics International is one such organization serving these remote areas. Pakistan must feel proud of its health workers who came forward to save the sick, fully aware of the dangers ahead.

To serve despite all risks, has to be called a virtuous act. While the governments have failed in fulfilling their duties to supply them with the necessary equipment’s, the doctors and nurses and paramedics have gone beyond the call of duty to save human lives.

Imagine a soldier being sent to a war front without the necessary arsenal. Or indeed a fire fighter heading to a towering inferno without tons of protective gear. It will never happen, and yet we see that our colleagues across the globe are fighting the battle against the CVID-19 without adequate equipment

British people applaud the NHS on Thursday evenings nationwide, a practice to be followed across the world. If you can’t give them due equipment at least give them a cheer.So let us compliment the health workers across the world, as they are virtuous people indeed.
